# Tropical cyclones cumulatively control regional carbon fluxes in Everglades mangrove wetlands (Florida, USA)

**DOI:** 10.1038/s41598-021-92899-1

**Published:** 2021-07-06

**Authors:** Xiaochen Zhao, Victor H. Rivera-Monroy, Luis M. Farfán, Henry Briceño, Edward Castañeda-Moya, Rafael Travieso, Evelyn E. Gaiser

**Affiliations:** 1 Department of Oceanography and Coastal Sciences, College of the Coast and Environment, Louisiana State University, Baton Rouge, LA 70803 USA; 2 Unidad La Paz, Centro de Investigación Científica y de Educación Superior de Ensenada, Baja California Sur, Mexico; 3 Institute of Environment, Florida International University, Miami, FL 33199 USA

**Keywords:** Ecology, Wetlands ecology, Carbon cycle

## Abstract

Mangroves are the most blue-carbon rich coastal wetlands contributing to the reduction of atmospheric CO_2_ through photosynthesis (sequestration) and high soil organic carbon (C) storage. Globally, mangroves are increasingly impacted by human and natural disturbances under climate warming, including pervasive pulsing tropical cyclones. However, there is limited information assessing cyclone’s functional role in regulating wetlands carbon cycling from annual to decadal scales. Here we show how cyclones with a wide range of integrated kinetic energy (IKE) impact C fluxes in the Everglades, a neotropical region with high cyclone landing frequency. Using long-term mangrove Net Primary Productivity (Litterfall, NPP_L_) data (2001–2018), we estimated cyclone-induced litterfall particulate organic C (litter-POC) export from mangroves to estuarine waters. Our analysis revealed that this lateral litter-POC flux (71–205 g C m^−2^ year^−1^)—currently unaccounted in global C budgets—is similar to C burial rates (69–157 g C m^−2^ year^−1^) and dissolved inorganic carbon (DIC, 61–229 g C m^−2^ year^−1^) export. We proposed a statistical model (PULITER) between IKE-based pulse index and NPP_L_ to determine cyclone’s impact on mangrove role as C sink or source. Including the cyclone’s functional role in regulating mangrove C fluxes is critical to developing local and regional climate change mitigation plans.

## Introduction

Blue carbon—the organic carbon sequestered and stored in coastal and marine ecosystems—is the most recognized ecosystem service worldwide^1–3^. This recognition is associated with the urgency to develop cost-effective mitigation measures to ameliorate climate change and variability, which are manifested by the increasing frequency of extreme events including excess precipitation and tropical cyclones^4–6^. Among blue carbon ecosystems, mangrove wetlands are the most productive^7,8^ and can significantly reduce carbon dioxide (CO_2_) in the atmosphere throughout photosynthesis (i.e., sequestration) and the accumulation of organic matter in the soil (i.e., storage)^9,10^. This accumulation is typically promoted by the pervasive anaerobic conditions—over thousands of years of geomorphological evolution—where mangroves are established due to regular and intermittent flooding occurring in a wide variety of coastal geomorphic settings across latitudinal gradients and climates^11–16^.

Indeed, mangrove wetlands are among “the most carbon-rich” ecosystems^8^ with a conspicuous carbon storage capacity, despite their limited extension and geographic distribution^17,18^ when compared to other blue carbon (i.e., seagrasses, marshes) and terrestrial (i.e., tropical/temperate/boreal forests, savanna and tundra, etc.) ecosystems^10^. It is estimated that mangrove wetlands globally store, in vegetation (above- and belowground) and soil compartments, approximately 4.19–6.17 Pg C^7,19^. During the last 10 years, this assessment has improved as more field data are acquired and through remote sensing tools at locations underrepresented in early attempts to produce a global mangrove carbon budget^20,21^. However, this value still has large uncertainties at the global scale, as unaccounted carbon budget can range from ~ 104–112 Tg C year^−1^^22,23^. Further improvements in this budget estimate are needed to implement feasible local and regional blue carbon conservation programs within the context of regional carbon markets, especially in countries situated in the Neotropics, Indo-Malayan, and Australasian ecoregions where most of the mangrove area is located^17,24,25^. This assessment is even more critical when attempting to assign an economic value to blue carbon as an ecosystem service given the major differences in mangroves physiognomic types (i.e., ecotypes)^26^, climate differences (e.g., arid vs tropical), water availability, and spatial distribution within a wide range of geomorphic settings (e.g., karstic vs deltaic)^11,13^.

In contrast to current global mangrove carbon storage estimates, carbon sequestration by both photosynthesis and soil accretion has been more elusive, especially where mangroves are undergoing severe and recurrent natural (i.e., tropical cyclones) and human disturbances (e.g., deforestation, urban development)^19,27,28^. One major challenge in estimating the net atmospheric carbon emission and capture in mangroves is the diversity of organic (e.g., particulate-POC, dissolved-DOC) and inorganic (dissolved-DIC, CO_2_, CH_4_) carbon (C) compounds that enter or exit coastal systems^22,29^. In contrast to terrestrial forests, mangrove productivity is not only regulated by nutrient availability that controls CO_2_ exchange (photosynthesis and respiration) between the forest and the atmosphere above the canopy (i.e., net ecosystem production, NEP), but also by hydrology affecting local hydroperiod^30–32^.

Hydroperiod—the frequency, duration, and depth of inundation—controls the lateral exchange of materials between mangrove wetlands and adjacent coastal waters, including the net exchange of both inorganic and organic carbon^7,33^. Yet, this lateral carbon flux magnitude is uncertain. Previous studies proposed that > 50% of the carbon fixed by mangrove vegetation is unaccounted (i.e., “missing carbon”)^22,23^ after counting potential carbon sinks (i.e., organic carbon export, sediment burial, and mineralization). This finding implies that in current mangrove carbon budgets, mineralization (e.g., decomposition and soil CO_2_ respiration) is significantly underestimated and therefore hypothesized that most of the carbon exported from mangroves to adjacent waters occurs as DIC^22^. Although few studies have been performed globally across different geomorphic settings and mangrove ecotypes to test that hypothesis, recent studies measuring DIC^34–40^, in addition to CO_2_ and methane (CH_4_) fluxes^41–49^, have partially confirmed the role of DIC, thus advancing our current understanding of the relative significance of these fluxes in regional carbon fluxes and budgets.

**Figure 1:**
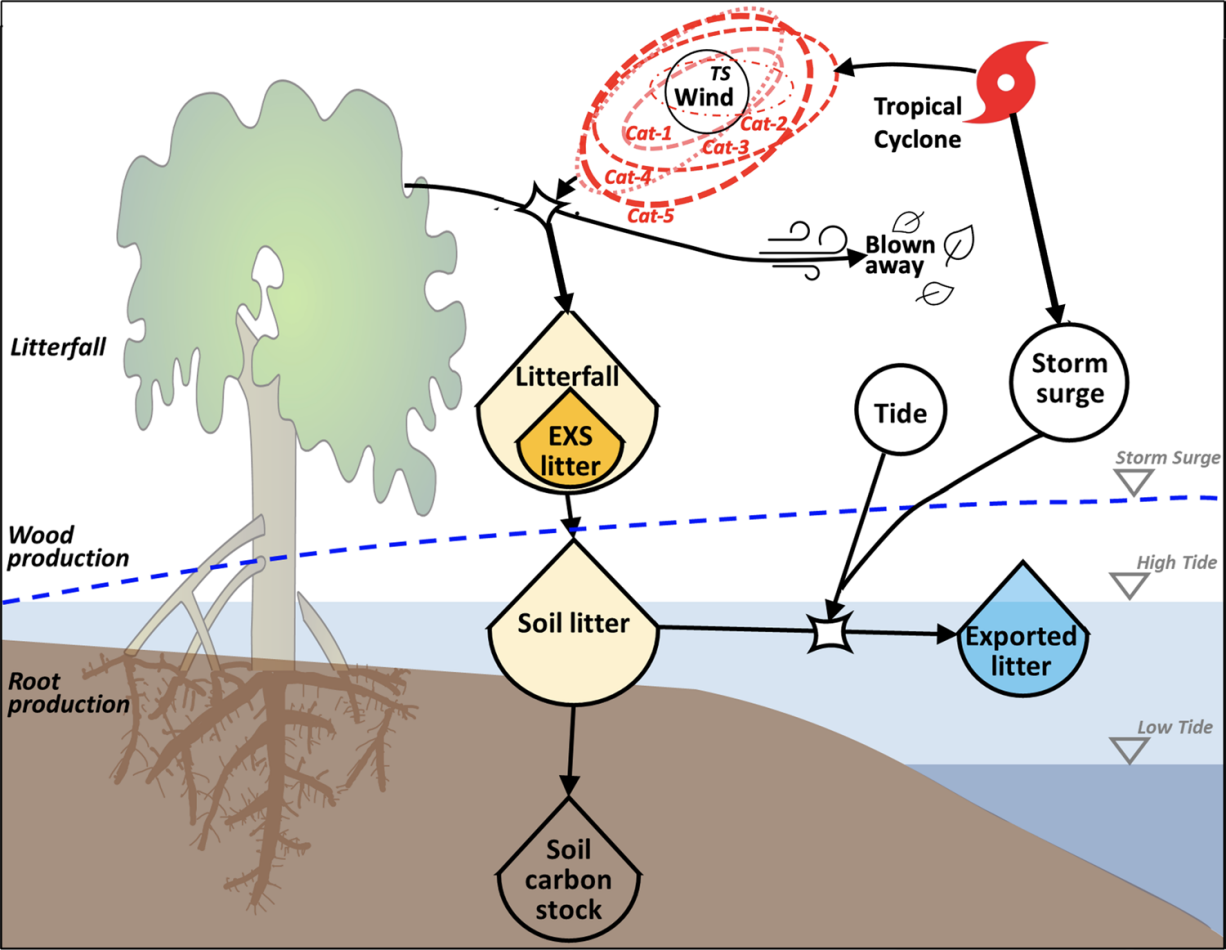
Conceptual diagram showing the impact of tropical cyclones on the mangrove forest canopy and hydrology along the Shark River Estuary. Wind velocity either causes different degrees of canopy defoliation or increases litterfall (i.e., EXS, excess litterfall) that accumulates in the soil (see Methods for EXS value estimation). Depending on the local hydroperiod, surface soil litterfall can be redistributed throughout the mangrove forest or exported to adjacent estuarine waters depending on the maximum water level and storm surge/tide duration. Cat-1, 2, 3, 4, 5 and TS (Tropical Storm) (Saffir-Simpson Hurricane Wind Scale) are storms with different Integrated Kinetic Energy (IKE) (terajoules; see Methods for explanation). Symbols are from Odum’s Energy Systems Language and the Integration and Application Network; https://www.osti.gov/biblio/5545893; https://ian.umces.edu/symbols).

Furthermore, another unaccounted flux in current global carbon budgets is the lateral flux of litter, i.e., Particulate Organic carbon flux (litter-POC) (Fig. 1), caused by tropical cyclones. This flux might contribute to the “missing carbon”^22^ in global mangrove budgets since it represents a knowledge gap when accounting for the fate of the forest total Net Primary Productivity (NPP_T_: litterfall, wood, and root production). This flux is apparent (i.e., “visible”^50^) since most cyclones are usually considered as major destructive, episodic events characterized by the Saffir-Simpson Hurricane Wind Scale (Categories 1–5; henceforth SSHWS National Hurricane Center: https://www.nhc.noaa.gov/aboutsshws.php)^51^. Hereafter, we used the term cyclone as equivalent to tropical cyclone that includes hurricanes (Cat-1, -2), major hurricanes (Cat-3, -4, -5) and tropical storms (TS). These pulsing events can cause high tree mortality and defoliation^52–60^ triggering subsequent long-term post-cyclone forest recovery in periods lasting from 5 to 10 years^44,61–65^. Moreover, cyclones with less destructive power can also impact forest structural and functional properties by regulating litterfall fluxes affecting organic matter accumulation in the soil in the short- or even long-term^66,67^ (Fig. 1). Assessing the role of this natural disturbance on mangrove carbon cycling will increase the scope and utility of economic assessments of blue carbon stocks when developing long-term climate mitigation programs under a warming climate that might intensify tropical cyclone frequency and intensity^2,3,5,10,68,69^.

The main objective of this study was to evaluate the functional role of cyclones in influencing the seasonal and interannual variation of litterfall organic carbon fluxes between the mangrove forest and adjacent coastal waters in the Shark River Estuary (SRE), South Florida. This subtropical estuary is in the northern Gulf of Mexico where cyclone landing frequency is high and with a wide range in energy content^70,71^. We developed a robust statistical model (henceforth PULITER) directly relating cyclone strength to the magnitude of “pulsing” litter deposition (henceforth EXS value) using the cyclone integrated kinetic energy (IKE) as an efficient metric of the destruction potential based on wind-field size and magnitude reported in energy units (from kilo- to terajoules)^72^. We hypothesized that the IKE is an efficient proxy to determine the magnitude of the “pulsing” excess litterfall net primary production (NPP_L_; i.e., EXS) at different cyclone energies.

The IKE differs from the SSHWS classification since the latter is based solely on the sustained wind speed on a scale of categories ranging from Cat-1 to Cat-5. In contrast, the IKE is a practical energy-based integrative measure of the destructive power at different spatiotemporal scales. The IKE can separate potential wind impacts based on cyclone trajectory while retaining the concise range of the SSHWS. To quantify the cyclone strength, we defined a new indicator—a pulse index (PI, see Eq. (2) in “Methods”)—comprising: (1) the IKE value; (2) the distance of mangrove study sites from the cyclone center (henceforth distance); and (3) the cyclone timespan (${t}_{S}$) covering the period when the distance is < 300 km from the mangrove forest.

Previously we have measured litterfall rates and their contribution to the NPP_T_ in three mangrove sites along the SRE over 18 years (2001–2018); we also evaluated the seasonal and interannual litterfall Net Primary Productivity (NPP_L_) variability during the presence and absence of cyclones of different strengths^62,65,73^. Using this information, we propose first-order estimates of the cyclone-induced EXS portion (i.e., litter-POC) that is exported to the adjacent estuary caused by the storm surge. Although NPP_L_ is one of the most extensive and well-documented organic carbon fluxes from the canopy to the soil in mangrove forests^74–78^, it is unknown how much of this flux contributes to the POC lateral exchange between the mangrove wetland and adjacent coastal waters before and after a cyclone impact. Finally, we compare these litter-POC export rates to other published DIC and DOC exchange rates measured in the same study sites to evaluate the overall carbon lateral flux as a result of cyclone impacts in the SRE and their significance to develop global mangrove carbon budgets.

## Results

### Cyclone events inducing defoliation and excess litterfall (EXS)

**Figure 2:**
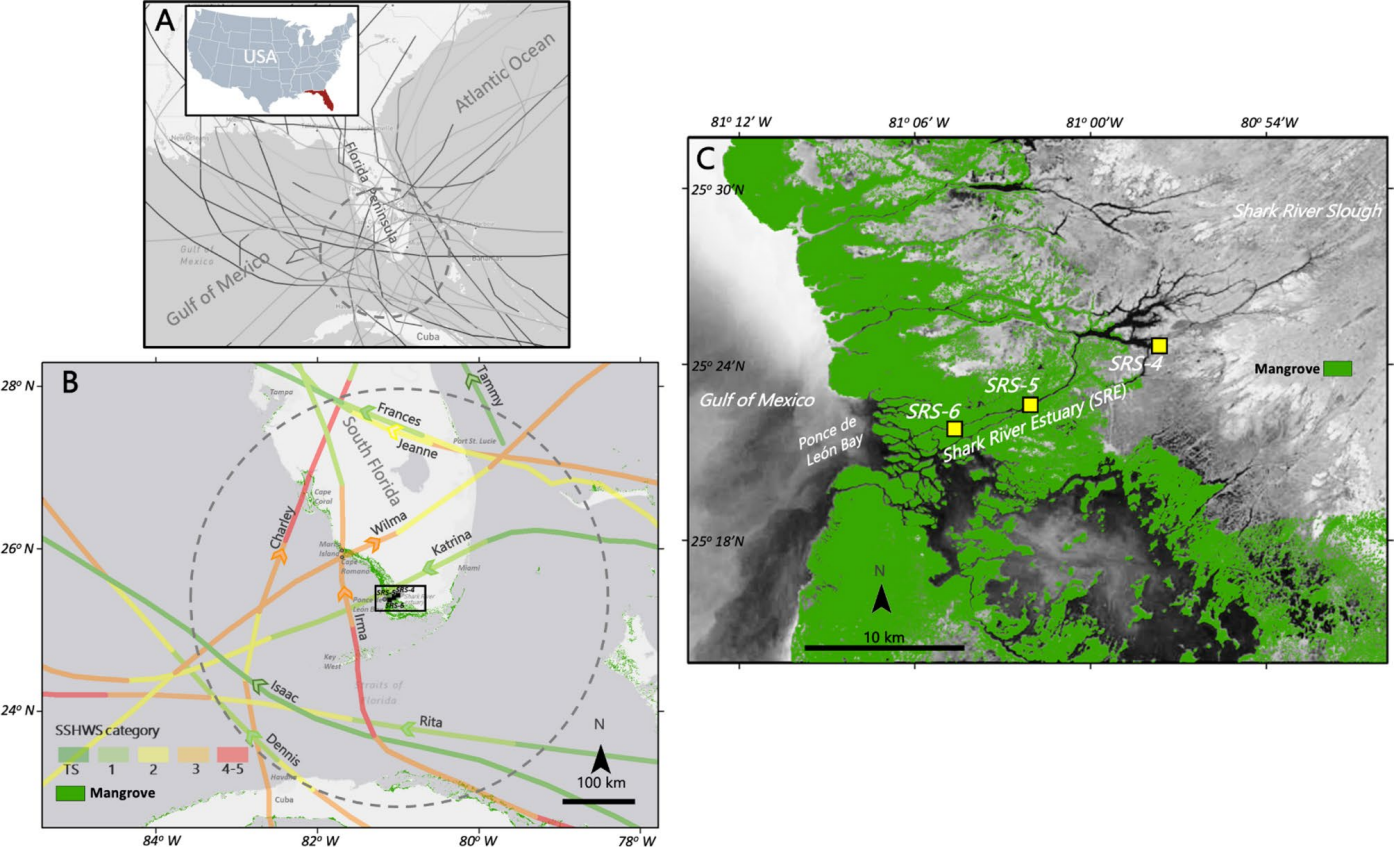
**(A**) Tropical cyclone trajectories (N = 28) in the Gulf of Mexico and Atlantic Ocean around the Florida Peninsula, USA from 1990–2018 including those that passed within a 300 km-radii (dotted circle center: Lat 25°22′37.20"N, Long 81° 1′55.20"W) from the mangrove study sites along the Shark River Estuary (SRE) in the Everglades, South Florida. (**B)** Selection of eight cyclones impacting study sites included in the analysis of Litterfall Net Primary Productivity (NNP_L_). Tropical cyclones Rita and Tammy are shown to indicate the relative impact on the study area depending on trajectory and distance to the study sites (see “Discussion”). (**C)** Location of mangrove study sites (SRS-4, SRS-5, SRS-6) along the Shark River Estuary (SRE). *SSHWS* Saffir-Simpson Hurricane Wind Scale. Mangrove coverage data is available as part of the Global Mangrove Watch (GMW) project^17^); track data is available from the National Hurricane Center (see “Methods” for details). Panels B and C were created using ArcGIS Desktop 10.8.1 mapping software.

Historically, twenty-eight cyclones made landfall or followed a parallel trajectory to the Florida coastline while passing within 300-km distance from our study sites in the 1990–2018 period (see Supplementary Table S1). Eighteen events occurred during 2001–2018, while ten were registered from 1990–2000 (Fig. 2A). Hurricanes Andrew (Cat-4, 1992), Wilma (Cat-3, 2005), and Irma (Cat-3, 2017) were major hurricanes and caused tree mortality and defoliation in the Florida Coastal Everglades (FCE) mangroves^52,57,59,60,62,65,79^.

The litterfall rate in the period 2001–2003, when no cyclones caused defoliation, was defined as the NPP_L_ baseline value and ranged from 830–1054 g m^−2^ year^−1^ (see Methods). Although during that period two weak cyclones developed in the Gulf of Mexico (TS: Gabrielle, 2001; Erika, 2003; Table S1), they moved outside the 300-km radius boundary from the Shark River Estuary (Fig. 2C) without causing any significant damage to our mangrove study sites. Afterward, the cyclone frequency increased in the FCE from 2004–2018. The maximum positive litterfall anomaly registered by comparing the annual litterfall rates in this period to the NPP_L_ baseline value (Fig. S1), ranged from 50–602 g m^−2^ year^−1^. In contrast, the maximum negative anomalies were observed immediately after cyclone passage with high IKE in 2006 (i.e., 469–716 g m^−2^ year^−1^) after Wilma (2005) and in 2018 after Irma (i.e., 96–741 g m^−2^ year^−1^) impacts.

Over the period of 2004–2018, eight cyclones caused considerable EXS (i.e., pulsing litter deposition) values: one Cat-4 hurricane (Charley, 2004); two Cat-3 hurricanes (Wilma, 2005; and Irma, 2017), four Cat-1 or -2 hurricanes (Frances and Jeanne, 2004; Dennis and Katrina, 2005) and one TS (Isaac, 2012) (Fig. 2B, Table S2). Overall, the cyclone-induced EXS ranged from 40.7 ± 5.8 to 940.9 ± 56.9 g m^−2^ per cyclone (N = 8) in the period from 2004 (Charley) to 2017 (Irma) (Table 1). EXS values associated with the highest energy events ranged from 487.7 (± 16.1) to 629.5 (± 38.1) g m^−2^ in the case of Wilma (R_EXS_: 58.9%, where R_EXS_ = EXS/annual NPP_L_ baseline; see Methods) and from 644.8 (± 74.9) to 940.9 (± 56.9) g m^−2^ after Irma impact (R_EXS_ range: 74.9–88.0%). The R_EXS_ values of other relatively low-energy cyclones ranged from 3.9 to 33.7% (Table 1).

**Table 1:** Physical properties of tropical cyclones impacting mangrove study sites (SRS-4, SRS-5 and SRS-6) along the Shark River Estuary, Everglades, South Florida, USA in the period 2004–2018, including associated litterfall values.

Year	Cyclone	Passage date	Study site	Litterfall sampling date	EXS (g m^−2^)	R_EXS_ (%)	Distance (km)	SSHWS	IKE_T_ (TJ)	IKE_Q_ (TJ)	Timespan (h)	PI_T_ (TJ km^−1^)	PI_Q_ (TJ km^−1^)
2004	Charley	13-Aug	SRS-4	16-Aug	69.6 ± 12.6	8.0	163.0	4	11.3	5.9	7.1	0.49	0.19
			SRS-5		61.2 ± 25.8	7.2	158.9	4	11.3	5.9	7.6	0.54	0.29
			SRS-6		40.7 ± 5.8	3.9	165.8	3	15.5	8.9	7.9	0.58	0.32
	Frances	5-Sep	SRS-6	NA	NA	NA	222.0	2	81.4	32.5	1.5	0.55	0.22
	Jeanne	26-Sep	SRS-6	13-Oct	100.6 ± 21.3	9.5	220.4	2	63.1	22.7	1.5	0.45	0.17
2005	Dennis	9-Jul	SRS-4	21-Jul	92.6 ± 33.4	10.8	267.5	1	53.1	37.2	3.0	0.60	0.42
			SRS-5		53.8 ± 36.6	6.2	260.0	1	53.1	37.2	3.0	0.61	0.43
			SRS-6		88.5 ± 25.9	8.1	255.6	1	53.1	37.2	3.0	0.62	0.44
	Katrina	26-Aug	SRS-4	13-Sep	293.7 ± 67.6	33.7	31.9	1	9.7	6.1	28.5	3.85	2.72
			SRS-5		50.3 ± 5.9	5.5	27.0	1	11.2	9.6	28.7	4.01	2.92
			SRS-6		240.6 ± 15.5	22.8	22.6	1	11.2	9.6	31.2	4.36	3.20
	Wilma	24-Oct	SRS-4	1-Nov	511.6 ± 36.4	58.9	87.9	2	121.0	72.9	8.1	7.56	4.40
			SRS-5		487.7 ± 16.1	58.9	91.6	2	121.0	72.9	8.2	7.72	4.51
			SRS-6		629.5 ± 38.1	58.9	86.2	3	116.6	70.9	8.3	7.91	4.64
2012	Isaac	27-Aug	SRS-6	10-Sep	104.7 ± 37.2	10.5	188.2	0	41.1	30.1	8.1	1.73	1.26
2017	Irma	10-Sep	SRS-4	10-Oct	644.8 ± 74.9	74.9	72.3	3	140.3	94.9	20.2	25.41	14.98
			SRS-5		655.4 ± 64.2	82.6	64.6	3	140.3	94.9	20.9	27.17	16.15
			SRS-6		940.9 ± 56.9	88.0	60.0	3	140.3	94.9	21.1	28.33	16.91

*EXS* total amount of excess litterfall material, *R*_*EXS*_ ratio of the EXS value to annual litterfall baseline (2001–2003), *IKE* integrated kinetic energy, *TJ* terajoules, *SSHWS *Saffir-Simpson Hurricane Wind Scale, *IKE*_*T*_ IKE value considering all cyclone quadrants (NE, NW, SE, SW), *IKE*_*Q*_ IKE value for selected cyclone quadrants, *PI* cumulative energy of a cyclone for a timespan and distance, *PI*_*T*_ PI value based on IKE_T_ for all quadrants, *PI*_*Q*_ PI value using IKE_Q_ for selected quadrants, “*NA*” not measured. Listed distance, SSHWS, IKE_T,_ and IKE_Q_ values were recorded when the cyclone was the closest to study sites. See “Methods” for further information and calculations.

### Tropical cyclone integrated kinetic energy and pulse index (PI)

**Figure 3:**
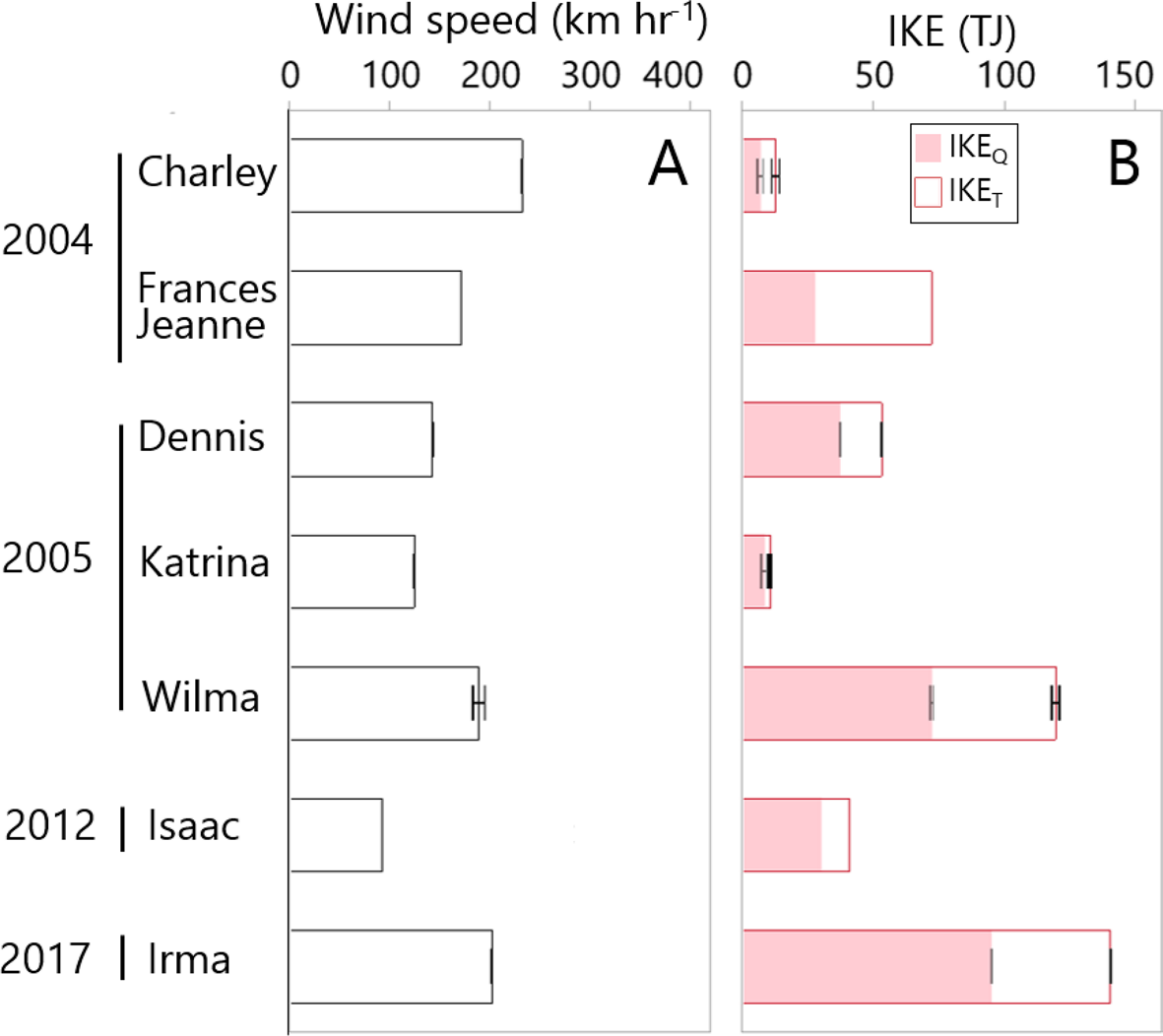
Means (± SE) of (**A)** wind speed (km h^−1^) and (**B)** Integrated Kinetic Energy (IKE; *TJ* terajoules) during the timespan when tropical cyclones impacted the Shark River mangrove study sites from 2004 to 2017. *IKE*_*Q*_ selected quadrants IKE, *IKE*_*T*_ all quadrants IKE; see “Methods” for IKE calculation.

Due to the interaction between cyclone wind circulation and path, each cyclone quadrant (see Methods) has different IKE values. When all quadrants are included (IKE_T_), the values ranged from low values (9.7–11.2 TJ; Katrina) to 140.3 TJ (Irma) (Table 1; Fig. 3). These values were estimated when each cyclone was close to our study sites (< 300 km). Irma and Wilma (IKE_T_: 116.6–121.0 TJ) were the most powerful cyclones followed by Frances (81.4 TJ), Jeanne (63.1 TJ) and Dennis (53.1 TJ). Interestingly, TS Isaac IKE_T_ (41.1 TJ) was more than three times over the value estimated for Hurricanes Katrina and Charley (9.7–15.5 TJ). Overall, the sub-quadrant IKE values of two selected quadrants (IKE_Q_) exhibited similar patterns as the IKE_T_ but with lower magnitudes (Table 1; Fig. 3).

The total Pulsing Index (PI_T_), defined as the cumulative energy of a cyclone during a timespan (see Methods) was calculated based on the IKE_T_ value and ranged from 0.45 (Jeanne) to 28.33 (Irma) TJ km^−1^ (Table 1). As expected, the quadrant PI (PI_Q_) was lower (0.17 to 16.91 TJ km^−1^; Table 1) when using the IKE_Q_ value. This index differs from the SSHWS where wind speed is the main criteria. Thus, based on the PI value, we categorized the cyclones included in this study as weak (PI_T_ < 2 TJ km^−1^; Charley, Frances, Jeanne, Dennis, Isaac), moderate (PI_T_ = 2–5 TJ km^−1^; Katrina) and strong (PI_T_ > 5 TJ km^−1^; Wilma and Irma).

### Pulse litter model (PULITER)

**Figure 4:**
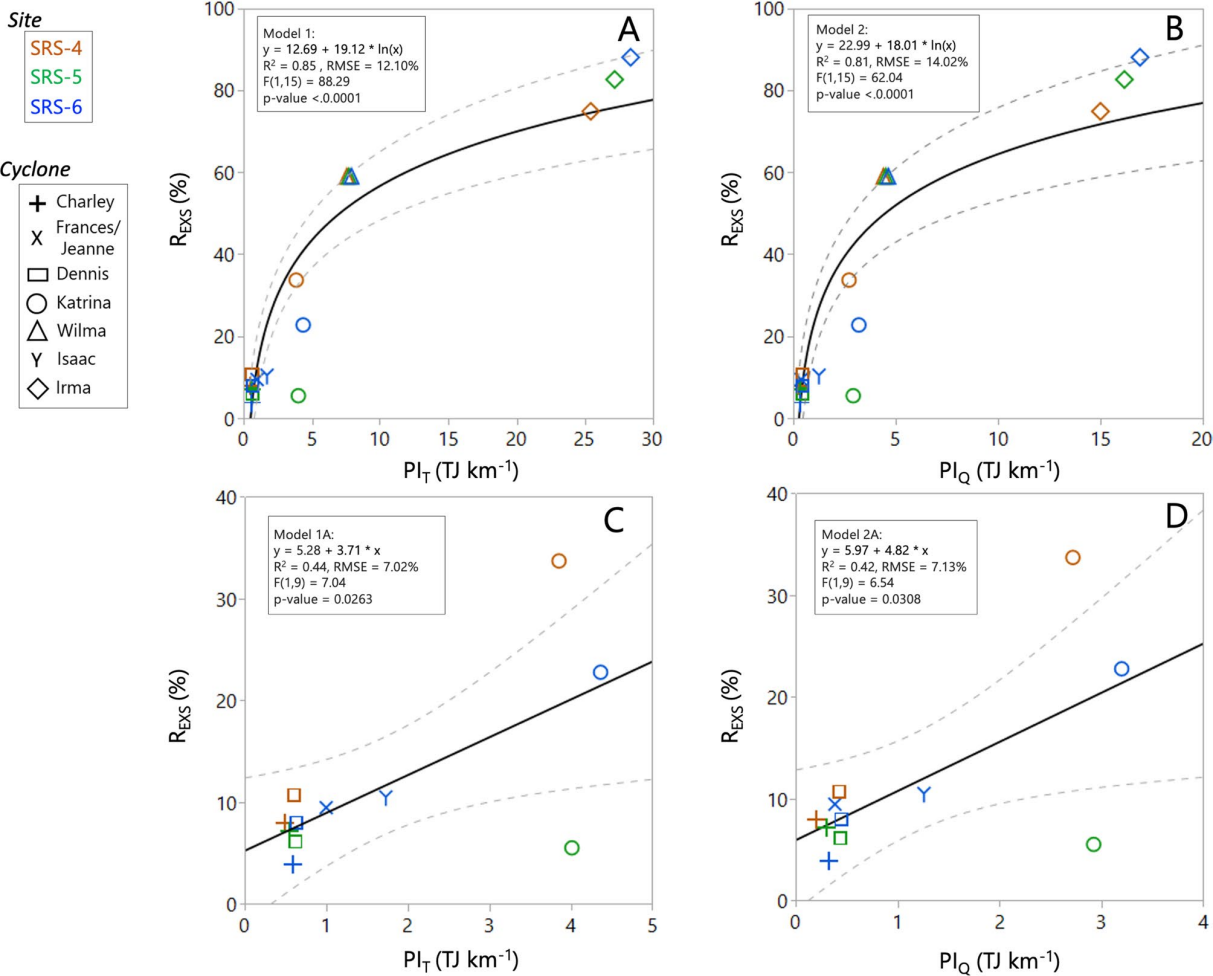
Non-linear and linear regressions between the R_EXS_ and pulsing index (PI) values (PULITER Model). (**A,C)** are plots between R_EXS_ and the PI_T_ value estimated using all the cyclone quadrants integrated kinetic energy (IKE) values (see “Methods” for details). (**B,D)** show the relationship using selected quadrants IKE when estimating the PI_Q_ value. Notice the change in the x-scale in **(C,D)** when strong cyclones Wilma (Category 3, 2005) and Irma (Category 3, 2017) were excluded from the regression analysis. The dashed curve indicates the 95% confidence interval.

Cyclones with higher IKE values were characterized by longer timespan and short distance as reflected by the high R_EXS_ values. We evaluated two non-linear statistical models to determine differences in the relationship between the R_EXS_ and PI_T_ (Model 1) and PI_Q_ (Model 2) (Fig. 4). The coefficient of determination (R^2^) was significant and high, with minor differences between Model 1 (R^2^ = 0.85; RMSE = 12.10%; p < 0.0001) and Model 2 (R^2^ = 0.81; RMSE = 14.02%; p < 0.0001) (Fig. 4A,B).

To further evaluate the effect of cyclone trajectory and direction, we only included weak and moderate cyclones and excluded Wilma (116.6–121.0 TJ) and Irma (140.3 TJ), which were strong cyclones (Table 1). This relationship was linear in both cases when using PI_T_ (Model 1A) and PI_Q_ (Model 2A) as independent variables (Fig. 4C,D). In both cases, the R^2^ value decreased to 0.44 (Model 1A: RMSE = 7.02%; p = 0.02) and 0.42 (Model 2A: RMSE = 7.13%; p = 0.03). This lower R^2^ is attributed to the highly variable R_EXS_ value registered among mangrove sites from Katrina where the impact was different among each study site due to the interaction between cyclone trajectory and speed (Fig. 2, Table S1). Still, these linear statistical relationships were significantly higher than when using only wind speed and distance as independent variables (Fig. S2).

### Litter export driven by tropical cyclone storm surge

**Figure 5:**
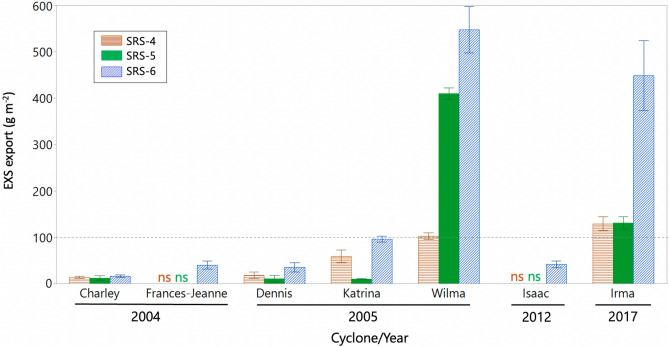
Mean (± SE) storm surge-induced EXS export values (excess litterfall) in three mangrove study sites (SRS-4, SRS-5, SRS-6) along the Shark River Estuary in the Everglades, Florida USA (see “Methods” for details in calculation and analysis). Notice the “ns” indicating no significant EXS values in SRS-4 and SRS-5 in the case of cyclones Frances-Jeanne (2004) and Isaac (2012). Dashed line indicates 100 g m^−2^.

Using the cyclone-induced excess litter (EXS) values (Table 1), we determined how much of this EXS was potentially exported through storm surges depending on the cyclone’s energy (see “Methods”; Fig. 5). In the case of Wilma, the exported EXS value was 547.6 ± 50.1 g m^−2^ at SRS-6 and 409.9 ± 12.2 g m^−2^ at SRS-5; these values represented ~ 49–52% of the annual NPP_L_ baseline determined at each site. At SRS-4, the exported EXS value was 102.3 ± 7.3 g m^−2^ and equivalent to 12% of the baseline. In the case of Irma, the exported EXS values in all sites were lower than in the case of Wilma: 448.3 ± 75.2 g m^−2^ (SRS-6), 131.1 ± 12.8 g m^−2^ (SRS-5), and 129.0 ± 15.0 g m^−2^ (SRS-4) (Fig. 5); these values represented approximately 43%, 16%, and 15% of the baseline, respectively. Weak and moderate cyclones, potentially, can cause an export range from ~ 10–96 g m^−2^ (below 100 g m^−2^; marked by a dashed line in Fig. 5). This exported range accounted for ~ 1–9% of the annual NPP_L_ baseline (830–1054 g m^−2^ year^−1^; lower boundary: 10/830 ≈ 1.2%; upper boundary: 96/1054 ≈ 9.1%).

These litter estimates were converted to carbon values to evaluate the particulate organic carbon export to adjacent coastal waters (litter-POC, see Methods). Overall, the litter-POC export was highest at SRS-6 (205 ± 23 g C m^−2^ year^−1^), followed by SRS-5 (84 ± 11 g C m^−2^ year^−1^), and SRS-4 (71 ± 5 g C m^−2^ year^−1^); these rates included the litterfall material exported through both tides and storm surge over the eighteen-year study period (2001–2018, N = 18; see Methods; Table 2). Overall, the litter-POC export showed an increasing trend along the estuary from upstream (SRS-4) to the mid- and downstream (SRS-5, SRS-6) estuarine regions. These values contributed to 10–24% of the mangrove NPP_T_ in our study sites (Table 2).

**Table 2:** Estimated carbon fluxes (g C m^−2^ year^−1^) (± SE) for riverine mangroves in the Shark River Estuary, Everglades, South Florida, USA. Values include total net primary productivity (NPP_T_ = litterfall + wood production + root production) and the fate (sensu Bouillon et al.^22^) of this organic matter.

NPP allocation (g C m^−2^ year^−1^)	SRS-4	SRS5	SRS-6	Median value		Source
Litterfall	357 ± 33.0	337 ± 23.3	446 ± 32.6	357		Castañeda-Moya et al.^73^; Danielson et al.^62^
Wood production	157 ± 18.0	64 ± 2.2	192 ± 12.8	157		Castañeda-Moya et al.^73^; Danielson et al.^62^; Rivera-Monroy et al.^65^
Root production	205 ± 28.6	283 ± 40.9	206 ± 41.8	206		Castañeda-Moya et al.^145^
NPP_T_	719	684	845	720		

Fate of NPP_T_ (g C m^-2^ year^-1^)					% of NPP_T_ accounted by the C flux	
POC export (tidal and storm surge)	71 ± 5	84 ± 11	205 ± 23	84	10–24	This study
Burial	69 ± 5.8	157 ± 13	123 ± 7.8	123	10–23	Breithaupt et al.^81^
Soil CO_2_ efflux	nd	nd	351^†^ (*)	351	42–51	Troxler et al.^49^
DOC export	nd	nd	56^§^ (*)	56	7–8	Romigh et al.^110^
DIC export	&	61–229^&^	&	145	7–34	Ho et al.^82^; Reithmaier et al.^83^; Volta et al.^42^
Total		608–964		759	85–128	

See “Methods” section for further information about calculations and assumptions. *SE values were no reported in the original source; *nd*  no data.

^†^Derived from in situ heterotrophic respiration measured over a range of flooding periods (inundated, partially exposed, and exposed).

^§^Obtained using flume measurements.

^&^Estimated using a combination of techniques: (a) in situ soil pore water data obtained at the SRS-5 site, (b) discrete sampling in the water column adjacent to the mangrove forest in all study sites and (c) longitudinal flux assessments using geochemical tracers along the Shark River Estuary.

## Discussion

Our analysis showed that the IKE is a valuable metric to operationally assess cyclone impacts with different energy content on mangrove forest structure, and to quantitatively evaluate the cumulative impacts on functional properties including NPP_L_ and ecosystem-level carbon storage. The advantage of this metric is the low number of parameters used to define its energy content^80^ and to identify energy differences among cyclone quadrants; this feature allowed a close examination of the spatially-explicit cyclone impacts. These quadrant-based IKE values (i.e., IKE_T_ or IKE_Q_) help partition the potential energy of wind fields across the landscape for mangrove forests in a post-disturbance analysis. Thus, IKE is a core component to define the pulse index (PI) that reflects the cumulative energy during a given period and distance (TJ km^−1^) from the mangrove forests.

The close association between PI and the excess litter values (i.e., R_EXS_, the temporal litterfall anomaly caused by a cyclone, see Methods) was high as stated in our hypothesis and described by the PULITER model (Fig. 4). This association can help to determine, not only the amount of litter-POC exported from the forest wetlands due to cyclones with variable kinetic energy, but also the relative contribution of the POC flux to the overall carbon budget as a lateral flux^22,23^ (Fig. 1). We found that cyclone-induced litter-POC export represents a considerable lateral flux that has not been explicitly included yet in mangrove forest global carbon budgets in coastal areas where tropical cyclones are a common seasonal and large-scale climatic disturbance impacting mangrove structure and productivity patterns (Fig. 1).

Further, our estimated litter-POC flux (range: 71–205 g C m^−2^ year^−1^) for mangrove forests in the SRE driven by both tides and cyclone storm surges was comparable to current estimates of soil carbon burial (69–157 g C m^−2^ year^−1^)^81^ and dissolved inorganic carbon (DIC) export (61–229 g C m^−2^ year^−1^)^42,82,83^ (Table 2). Generally, the POC flux included in global mangrove carbon budgets represents only the exchange between the forest and the adjacent coastal waters modulated by tidal exchange/hydrology but not cyclones^7,22^. Below we discussed the strength and caveats of the PULITER model and our proposed first-order litter-POC rates in the context of our current understanding of mangrove carbon cycling in the Everglades, and the ecological implications of this proposed cyclone-induced lateral carbon flux when attempting to constraint global mangrove carbon budgets.

### Pulse index and PULITER model

Although several linear and non-linear models have been developed to assess mangrove forest structural damage (e.g., area/basal area loss) after cyclone impacts using one or two meteorological variables (i.e., wind speed and distance)^63,84^, it is difficult to use cyclone strength properties to quantify functional mangrove properties^50,85^. We analyzed our R_EXS_ values against a set of typical cyclone strength variables (i.e., SSHWS, wind speed, distance, wind speed weighed by distance), and found that these relationships have a weak association accounting for only a small portion of the total variance (R^2^ range: < 0.1–0.3; Fig. S2). For example, although Charley (2004) was a Cat-4 cyclone with a high wind speed (231 km hr^−1^; Table S1) when it moved close to our mangrove study sites (Fig. 2), wind speed was inversely related to the R_EXS_ (Fig. S2). In contrast, the PI value classified Charley as a weak cyclone (PI_T_: 0.49–0.58 TJ km^−1^; Table 1) thus matching the observed low R_EXS_ values in all mangrove study sites. Therefore, using the PI value as an independent variable in the PULITER model (Fig. 4) significantly improves the coefficient of determination reflecting actual cyclone relative impacts on mangrove functional variables (i.e., NPP_L_).

Previous studies have shown how the interaction between cyclone wind speed and trajectory determines the magnitude in mangrove mortality and defoliation (i.e., Hurricane Andrew, Cat-4, 1992)^52,86^. The PI value used in the PULITER model defines this interaction directly and at a finer spatial scale by using either the total energy of the four quadrants (PI_T_) or the energy of two quadrants (PI_Q_) (Fig. 4). This partition helps to differentiate the relative cyclone impact depending on the cyclone trajectory and direction relative to the mangrove location (Fig. 4B, Model 2). Although the relatively short distance (< 15 km) among our three study sites along Shark River Estuary (Fig. 2B) limits the site-specific cyclone impact characterization (i.e., energy, distance, and duration) in comparison with the wind field radii (hundreds of kilometers), we still were able to determine differences in R_EXS_ values using the PI_Q_ value (Fig. 4B). Hurricane Wilma, for instance, coming from the Gulf of Mexico, made landfall as a Cat-3 on Cape Romano in the Florida Peninsula (Fig. 2C). This landing location was ~ 10 km from the location where Irma landed (Marco Island) as Cat-3 before moving across the peninsula towards the Atlantic coast. The shortest distance from Wilma’s landing site to our study sites was ~ 85 km. In contrast, Irma trajectory was parallel to the southwest Florida coastline passing closer (~ 60–70 km) to the same sites (Fig. 2B). This difference in cyclone trajectory resulted in different impacts: Wilma caused a 3-m storm surge^87,88^ while Irma’s storm surge was lower (~ 1 m)^55,89^ despite having PI_T_ ~ 3.5 × higher cumulative kinetic energy (Table 1). Hence, we estimated that the Wilma-induced storm surge exported > 80% of the litterfall towards the SRE down- and midstream regions, while Irma’s storm surge exported only 46% downstream closer to the SRE mouth (Fig. 5; see Methods).

Another example of the complex and wide range of cyclone impacts based on the interaction among cyclone trajectory, landing location, distance from study sites, and quadrant-energy content is the case of Cat-2 Hurricanes Frances and Jeanne in 2004 (Table 1). Both cyclones came from the Atlantic coast and made landfall north of the study sites (Fig. 2B). Although this spatial trajectory was expected to have a stronger impact on the EXS value at the upstream site, significant EXS values were measured only in the downstream (SRS-6) sites (Student’s t-test p < 0.05, Table S2). This site difference is directly related to the uneven cyclone quadrant energy content (Fig. S3) where both hurricanes had greater IKE values in the northeast (NE) and northwest (NW) quadrants. Since the wind circulation is counterclockwise in the Northern Hemisphere, the relatively high speed in the NE and NW quadrants had a stronger impact at the downstream mangrove location relative to the upstream SRS-4 site, as the cyclone moved along a North-Western trajectory (Fig. 2B), thus causing greater defoliation downstream.

The timespan (${t}_{S}$) variable included in the calculation of PI is also a critical component influencing the degree of cyclone impact on mangrove forest structure and productivity; especially when assessing low-energy (weak and moderate) cyclones. A weak cyclone cannot fully defoliate a forest canopy within a short period, yet it can do so when the wind speed is sustained over a long period causing litter accumulation on the forest floor. In contrast, a stronger cyclone can fully defoliate the forest canopy within few hours, not only contributing to soil litter accumulation but also “blown away” lighter litter material (e.g., leaves) out of the forest (Fig. 1). The extreme defoliation cases are depicted in our empirical PULITER model including a “tipping point” or transition where a maximum litterfall is observed at moderate PI values (i.e., Katrina) as represented in Models 1 and 2 when canopy defoliation is > 40–50% (Fig. 4). In this relationship, maximum R_EXS_ values are determined by the initial forest structure, particularly tree height and density, since taller trees are more susceptible to damage by variable wind speed^90–94^.

This pattern in cyclone timespan and trajectory is exemplified when comparing Katrina’s and Irma’s PI magnitude. Although Katrina’s PI value was ~ 15% of Irma’s, Katrina’s R_EXS_ values ranged from ~ 25–45% at both up- and downstream estuarine regions when compared to Irma’s values (Table 1). Katrina moved along a latitudinal path from the Atlantic coast to the Gulf coast across South Florida and passed close to our mangrove sites (~ 22–32 km) in contrast to Irma, which moved northward and parallel to the western Florida coastline (Fig. 2B). Because of the short distance and close IKE values, Katrina’s PI_T_ values were similar at SRS-5 (4.01 TJ km^−1^) and SRS-4 (3.85 TJ km^−1^) and slightly higher at SRS-6 (4.36 TJ km^−1^); however, the R_EXS_ at SRS-5 (5.5%) was lower than values at SRS-6 (22.8%) and SRS-4 (33.7%). This comparative analysis among cyclones highlights how a cyclone trajectory and distance can have different impacts on forest canopy, especially considering the distinct forest structures (e.g., tree height, species dominance) along the estuary^62,65^. Although Katrina-induced EXS at SRS-5 is not significantly different from zero (Table S2), this value was included in the PULITER model to represent how mangrove forest structure and distance interact in defining cyclone energy (PI) variability and thus determining R_EXS_ values. This result highlights how mangrove forest initial structure along the SRE (e.g., species composition and tree density, see “Methods”) can contribute to the observed differences in carbon fluxes.

### Cumulative tropical cyclone impacts and litter export

It is likely that cyclone strength and frequency in the North Atlantic Ocean basin will increase in the next decades^4,6,79,95,96^. Hence, we expect an increase in EXS values, and consequently variable litter-POC export (Fig. 5). This increasing trend in cyclone frequency in a single hurricane season was observed in 2005 and more recently in 2020 in the northern Gulf of Mexico^71^. In 2005, an array of cyclones with variable strengths struck South Florida underscoring the storm seasonal and variable cumulative impact across sites (Cat-1: Dennis, July 9th, and Katrina, August 26th; TS: Tammy, October 5th; Cat-2: Rita, September 20th and Wilma, October 24th) (Fig. 2B, Table S1). In that season, Katrina was a long-duration, moderate cyclone, that caused significant litter deposition (i.e., EXS) (Table 1). Twenty-five days after Katrina’s impact, Rita was in transit throughout the Strait of Florida, approximately 170 km south from our study sites (Fig. 2B), and then followed by Tammy, which formed close to Port St. Lucie, on the Florida Atlantic coast approximately 250 km from our study sites. In contrast to Katrina, the first month litterfall sample collection after Rita and Tammy (October 11th, 2005; no sample was collected between the occurrence of Rita and Tammy) did not show a significant EXS increase. This value reflected a litterfall deficit when compared to the monthly baseline values (range: − 57.3 ± 9.7 g m^−2^ to − 25.9 ± 9.6 g m^−2^) (Table S2), indicating that the forest canopy had not recovered to pre-Katrina conditions. Further, Wilma—with high energy content (120 TJ)—followed Tammy nineteen days later causing significant EXS values (R_EXS_ = 58.9%) across all mangrove sites. Based on these values, we estimated that > 80% of the litterfall was exported from the forest in the case of Wilma (Fig. 5). Cumulatively, the total EXS values caused by Dennis, Katrina and Wilma ranged from 592–959 g m^−2^ (i.e., R_EXS_ range: ~ 71–103%) (Table 1); these EXS values were slightly higher than those estimated for Irma’s impact in 2017. In fact, the short interval between the impact by Katrina (moderate) and Wilma (strong) was not long enough (~ 2 months) for the forest canopy to fully recover to pre-cyclone levels after Katrina impact^62^. Overall, this comparison highlights the utility of long-term studies to quantitively assess forest canopy resiliency^62,65^.

Globally, the estimated percentage of litter material exported from mangrove forests varies widely (range: 0–99%, mean: 51%)^74^. Currently, this wide range does not explicitly include the contribution of cyclones impact and it is partially explained by the strong interactions of a number of environmental process and variables including geomorphology, hydrology (e.g., drag coefficient interacting with hydrodynamic processes)^88,97–100^, the relative dominance of different ecotypes within a given geomorphic setting^11,26^, and local hydroperiod^30,101^. Since the actual tidal range in mangrove wetlands is also partially dependent on the wetland relative elevation and net freshwater discharge^32,102^, the tidal exchange between the wetland and the adjacent coastal water can vary even within the same geomorphic setting.

In South Florida’s karstic environment, the tidal amplitude along the SRE where our sites are located is ~ 0.75 m and different from values in other mangrove ecotypes ( e.g., basin 0.1 m; Table S3)^74^. The litter-POC flux in those Florida mangrove sites ranged from 186–438 g C m^−2^ year^−1^ representing up to 75–93% of NPP_L_. In contrast, our estimated litter-POC export is a relatively conservative value as we assumed 20% or 40% of the litter is exported to coastal waters under normal tidal cycles and weak cyclone-induced storm surge (see Methods). Our first-order range accounts for 19–44% of the annual NPP_L_ and is relatively lower than those reported for other sites in Florida mangroves including the average global value (51%)^74^ (Table S3). Hence, further direct measurements of this POC flux driven by the tidal exchange on a seasonal and annual basis can improve present and future variations in the EXS estimates.

The extreme condition when assessing EXS values is represented by a destructive cyclone impact that results in complete defoliation, tree snapping, and uprooting^93^ such as in the case of high-IKE cyclones (e.g., Wilma and Irma IKE: 121–140 TJ). As a result, the EXS export from the forest is generally > 40–80% of the observed EXS (Fig. 5) representing up to ~ 50% of the annual NPP_L_ baseline, in contrast to the low exported EXS (1–9%) under other low-energy cyclones (Table 1). Collectively, among the total eighteen cyclones occurring close to our study sites in the period 2001–2018, eight of them caused a wide range of exported EXS values (Fig. 5); in terms of carbon mass, this litter-POC is equivalent to an average range of 8–30 g C m^−2^ year^−1^ over the period 2001–2018 (N = 18 years) or 18–67 g C m^−2^ per cyclone (N = 8 cyclones).

The cumulative cyclone impact on annual litter deposition (i.e., EXS) and export must be considered in the long term (Fig. 1), not only in terms of cyclone frequency and intensity, but also with its interaction with sea level rise (SLR). Currently, South Florida’s SLR rate is among the highest over the last decade in the northern Gulf of Mexico (9 ± 4 mm year^−1^; 3 × the average pre-2006 rate, 3 ± 2 mm year^−1^)^103,104^. It is expected that this rate will continue to increase because of climate change causing significant alterations in regional hydrology and local hydroperiod in our mangrove sites^32,101^. Due to the location of each sampling site along the estuary (Fig. 2C), both frequency and duration of inundation are significantly different^32,73^. Therefore, we expect increasing export of litterfall accumulated on the forest floor in the next decade, especially when more frequent low-energy cyclones impact the mangroves under climate change^4,71,80,105^.

### Mangrove carbon fluxes and budget

Although several studies emphasize the importance of tropical cyclones on carbon cycling in mangrove forests, this carbon flux is not explicitly included in current mangrove carbon budgets^22,23^. This absence is partially due to the difficulty in evaluating cyclones’ impact at different spatiotemporal scales and the lack of long-term data before and after cyclone impact. Despite some limitations due to the number of cyclones with different IKE values, we were able to determine their impact on litterfall (i.e., EXS)—one of the major contributors to ecosystem NPP_T_^7,22,77^. The availability of a long-term litterfall record since 2001^62,73^ allowed us to capture the role of cyclone impact under different intensities. In addition to the lack of inclusion of this flux (EXS) in carbon budgets, there is a large uncertainty in other carbon flux estimates since most of the data used to construct carbon budgets is from different sites^7,13,22,106^. This approach is currently used since there are not enough mangrove sites with key information about carbon stocks and fluxes needed to implement a meta-analysis across different regions and climates^7,22,23^. Consequently, regardless of the accuracy and precision of each flux and stock data, current budgets are confounded by the intrinsic spatiotemporal variability given the differences in biogeochemical cycling among mangrove ecotypes with different spatial extent and distribution within a range of geomorphic settings^13,16,20,107^.

In the frequently cyclone-impacted Everglades karstic environment^108^, early estimates using the eddy covariance method and a mass balance approach showed that in Shark River’s riverine mangrove (SRS-6), the Net Ecosystem Carbon Balance (NECB) was 1038 ± 88 g C m^−2^ year^−1^ while the Net Ecosystem Exchange of CO_2_ with the atmosphere (-NEE) was 1170 ± 127 g C m^−2^ year^−1^^109^. Specifically in that mass balance calculation^109^, the overall net carbon lateral flux (F_TOT_ = -131 ± 155 g C m^−2^ year^−1^) was first estimated for the SRE by difference between those two fluxes (i.e., NECB = –NEE + F_TOT_). The F_TOT_ value calculation a priori included different carbon forms (i.e., DIC, DOC, POC, carbon monoxide, methane (CH_4_) and volatile organic carbon) and then compared to available direct measurements of selected carbon forms (DIC, DOC, POC) to constraint both the flux magnitude and uncertainty. However, partitioning the net carbon lateral flux (i.e., F_TOT_) for validation purposes was difficult since in situ measurements for several carbon forms are not available for the SRE^106^.

Yet, although limited, the available combined specific carbon fluxes can help constraint their overall magnitude and contribution to the total carbon lateral flux in the SRE (Fig. 1, Table 2). These include DOC measurements based on in situ sampling in the SRE downstream (SRS-6: 56 g C m^−2^ year^−1^)^110^ and POC values obtained in other locations in South Florida (64–186 g C m^−2^ year^−1^)^78,111^. This POC flux, for instance, compares to our estimated litter-POC flux range (tidal exchange + storm surge = 71–205 g C m^−2^ year^−1^). In the case of DIC, recent seasonal tracer release experiments have identified a range from 61–229 g C m^−2^ year^−1^ along the SRE^42,82,83^. The total sum of DOC + POC + DIC (Table 2; range: 188–490 g C m^−2^ year^−1^) using these field-based estimates is higher than the mass balance based on the F_TOT_ value (i.e., 131 ± 155 g C m^−2^ year^−1^)^ 109^) in previous studies. Interestingly, the range of field-based DIC measurements is much lower than the range used in other studies (i.e., 170–560 g C m^−2^ year^−1^) when attempting to determine the fate of the NPP_T_ using both the mass balance and eddy covariance methods^43,44^. Additionally, in situ soil CO_2_ efflux measurements at SRS-6 show that soil respiration over a range of flooding stages is 351 g C m^−2^ year^−1^ (Table 2)^49^. Because of the lack of a CH_4_ flux estimates in our mangrove study sites, this flux is not included in our analysis. Further studies are needed to determine CH_4_ net flux in different mangrove types and ecogeomorphic settings to account for this critical flux in global carbon budgets; especially given its higher radiative forcing compared to CO_2_^48^.

As mentioned, previous studies have hypothesized that DIC export might account for a large proportion of the “missing carbon” (> 50% of the total NPP_T_) in current global carbon budgets. In the case of the SRE, we found that the DIC flux represented 7–34% of the total NPP_T_ while POC accounted for 10–24%; these fluxes together represent 17–58% of the total NPP_T_ (Table 2). Our low litter-based POC value (i.e., 71 ± 5 g C m^−2^ year^−1^) is lower than that included in the global POC range (137–203 g C m^−2^ year^−1^)^7,22,112^ while our high estimate (205 ± 23 g C m^−2^ year^−1^) is closer to the maximum value in that range. This first-order calculation suggests that our POC flux, caused by the pulse cyclones with variable energy, can explain up to 24% of the missing carbon in this karstic coastal environment. This information can help constraint regional carbon budgets in neotropical mangrove wetlands impacted by increasing seasonal cyclone activity^105,113,114^.

When partitioning the NPP_T_ fate in the SRE, we discovered the similarity between the litter-POC later flux partially driven by cyclones and the carbon burial rates (69–157 g C m^−2^ year^−1^; Table 2) measured in the same study sites. If this range occurs across different mangrove ecotypes, then it could be assumed that the sink (i.e., burial) versus source (i.e., export) values should balance in the long term. Yet, given the pulsing nature of litter-POC that can be exported not only by the storm surge but also directly “blown away” from the forest canopy (Fig. 1), we expect a greater POC flux especially under the potential increase of weak and moderate cyclones (PI_T_: < 5 TJ km^−1^). Because of the inability to presently account for the “blown away” POC portion caused by extreme wind velocity, we consider our values as conservative estimates^62,65^ .

It is expected that in mangrove-dominated coastal areas impacted by cyclones, this large litter mass transported by strong winds and storm surge might end accumulating in different stages of decomposition at the bottom of shallow waters in creeks and embayment of deltaic or karstic coasts^67,115^—as is the case of South Florida. Presently, we do not know the magnitude of this accumulation and spatial distribution along the SRE and coastal waters (e.g., Ponce de León Bay, Fig. 2C). Certainly, this cyclone-induced net POC flux needs to be included when determining the relative importance of “outwelling”^116^ of both inorganic and organic carbon that contribute to primary and secondary production in coastal waters^117–119^. We hypothesize that the large extensions of this accumulated organic carbon reach tens of kilometers in shallow waters as indirectly shown by the cyclone wind field patterns across the SRE and in other coastal regions in the Gulf of Mexico. Further, groundwater (i.e., submarine discharge) in Florida’s karstic environment also plays a major role in the export of dissolved inorganic/organic carbon and inorganic nutrient to the coastal zone requiring more field-based measurements in this region^102,120,121^.

### The role of mangrove recovery and resilience in long-term carbon storage and sequestration

Mangrove plant adaptations are manifested on the species-specific response to major disturbances including resistance to windstorm. It is reported that *Rhizophora* *mangl*e is more susceptible to wind damage than *Avicennia* *germinans*^63^. Indeed, wind velocity has different effects on vegetation depending on the variable leaf angle, canopy geometry and canopy height spatial distribution^122–124^ that can directly influence the magnitude of EXS values. The difference in species composition along with species-specific resistance to wind impact from the same cyclone could explain litterfall differences among sites.

Although few studies have directly measured mangrove canopy trait responses to wind impacts, modeling studies have explicitly simulated forest canopy geometry, demonstrating the importance of crown geometry when trees compete for resources (e.g., light, nutrients) before and after cyclone impact^125–127^. For instance, *Laguncularia* *racemosa* dominance in forest stands located close to the SRE mouth (i.e., SRS-6) highlights the recurrent impact of cyclones where this species growth rate and reproductive output are enhanced when light availability increase after defoliation or tree mortality causes large forest gaps^65,73,124,128^. This species dominance is further enhanced when soil P limitation is offset by the landscape-level inorganic P inputs by sediment deposition driven by storm surges^89^. As a result of this P deposition, mangrove forests in the SRE recovered quickly within five years after Wilma’s impact, which caused > 90% defoliation and low cumulative tree mortality (< 16%) post-disturbance^62^. Under this scenario, e.g., Wilma’s impact^62^, trees are expected to allocate more energy to foliage production for expedited refoliation^129–132^, hence potentially arresting stem growth (e.g., tree height) and development^133,134^ that can alter both carbon sequestration and storage rates in the short (1–3 years) and mid-term (decades) periods.

Finally, to assess the relative role of mangrove forests as carbon sinks or sources at the global scale, and comparatively to other blue-carbon wetlands, we recommend not only describing the site geographical features but also its ecogeomorphic setting, mangrove ecotype(s) spatial extension—and critically—the frequency of cyclone impacts along the coastline^122,135^. As previously reported^20^, mangrove tree height and aboveground carbon stocks in cyclone-prone regions are globally lower than those in coastal areas not impacted by tropical cyclones. This difference in carbon allocation at the regional scale (> 10 km^2^) has major implications in the amount of carbon storage and net carbon export in the long term^106^. This geographic and latitudinal distinction is needed when determining—not only current and future blue carbon budgets per ecoregion and country— but also their economic value in climate change mitigation plans as carbon markets are developed and implemented within the next decade^2,3,10,68,69^.

## Methods

### Study sites

The Everglades mangrove wetlands are in the southern region of the Florida Peninsula, Florida (USA). This peninsula intersects the trajectory of highly frequent tropical cyclones in the Gulf of Mexico (Fig. 2). As part of the Florida Coastal Everglades Long-Term Ecological Research (FCE LTER) network^136^, three mangrove study sites were established along Shark River Estuary (SRE) with different distance from the mouth of the estuary: 4.1 km (SRS-6), 9.9 km (SRS-5) and 18.2 km (SRS-4) (Fig. 2C). The mangrove species *Rhizophora* *mangl*e, *Laguncularia* *racemosa* and *Avicennia* *germinans* are present at SRS-6 and SRS-5; *A.* *germinans* is replaced by the mangrove associate *Conocarpus* *erectus* at SRS-4 located at the mangrove-marsh ecotone boundary. These sites show distinct gradients in hydroperiod (i.e., inundation duration, frequency, and depth), soil nutrient (e.g., phosphorus) and stressors (e.g., salinity and sulfide)^62,73,137^. Tree height decreases (~ 18 to < 6 m) from downstream (SRS-6) to upstream (SRS-4)^20,138^; basal area ranges from ~ 40–20 m^2^ ha^−1^ and tree density increases (2838–7746 trees ha^−1^) from downstream to mid- (SRS-5) and upstream estuarine regions^73,137^. Two 20 m × 20 m permanent plots were established at each site and five wooden baskets (N = 10) were placed at random locations in each plot following standard sampling litterfall techniques^106^. The baskets are 0.5 m × 0.5 m and lined with 1- mm meshed screen and standing at ~ 1.5 m height above the forest floor. Monthly litterfall samples were collected in paper bags and transferred to the lab for further analysis. Litterfall samples were oven-dried at 60 °C for 72 h and then sorted into different components, including leaves, reproductive materials (i.e., propagules, flowers) per species, and twigs/wood. The dry weight was recorded within 0.01 g for each component^62,73^. A conversion factor of 0.44 was used to convert dry mass (g m^−2^) to carbon content (g C m^−2^)^139,140^.

### Tropical cyclone frequency (2001–2018)

The northern Gulf of Mexico, including southern Florida, is directly and indirectly impacted by tropical cyclones originated in the North Atlantic Ocean and the Gulf of Mexico. The seasonal number of cyclones is variable and with different degrees of intensity gauged by wind speed; the lifetime maximum intensity typically occurs in the Gulf of Mexico basin^71^. In extreme cases, they can cross the Florida Peninsula or pass in parallel to the coastline within one to hundreds of kilometers^108,141^. In this study, we identify potential impact over our study sites, by first identifying cyclones registered from 2001 through 2018 (https://coast.noaa.gov/hurricanes), and then selecting events that could impact our mangrove study sites within the period when Litterfall Net Primary Productivity (NPP_L_) data was available. Thus, we delimited a circular area with the center reference point established at the midstream study site (SRS-5; 25°22′37.20"N, 81° 1′55.20"W) with a 300-km radius (Fig. 2B). This distance was selected to warrant detection of the cyclone determined, initially, by wind speed and category as listed in the historical dataset.

### Integrated kinetic energy (IKE) and pulsing index (PI)

Tropical cyclone structure is complex due to the compound effect of variable components including wind speed, circulation size, trajectory, and translation motion. Integrated kinetic energy (IKE)^72^ determines the cyclone energy comprising wind speed and horizontal length:

1 $$IKE\,=\,{\int }_{V}\frac{1}{2}\rho {U}^{2}dV$$

where $\rho$ is the air density, 1.15 kg m^-3^; U is the surface wind speeds within specific ranges (low, moderate, and high speed); V is the volume of the storm domain. For each cyclone, we calculated the IKE for each quadrant. The sum of IKE for these four quadrants becomes the total IKE (IKE_T_). We also identified two quadrants that the study sites were inside or close to, and then defined the sum of IKE for these two quadrants as sub-quadrant IKE value (IKE_Q_).

We identified times when the cyclones moved near to the study sites within 300-km along the track and calculated the quadrant and total IKE using an on-line tool (https://www.aoml.noaa.gov/hrd/ike/Calculator_AllQuad.php). Because the historical dataset was reported in six-hour intervals, we performed a linear interpolation to estimate IKE values for short intervals (1–3 h) to be consistent with the track records (https://www.nhc.noaa.gov/data/hurdat/hurdat2-1851-2019-052520.txt).

We considered the timespan (${t}_{S}$) as the period when the mangrove area is impacted by high winds as the cyclone center is within 200-km range from the study sites. In the case of cyclones passing beyond 200 km, but within a 300-km range, we only counted the hours when the cyclone center was the closest (Table 1). Along that specific trajectory segment, $i$, we assumed that the IKE (TJ) values (${IKE}^{i}$) and distance (${Distance}^{i}$) were constant during the transit timespan, ${t}_{S}^{i}$. Using these terms, we defined the pulsing index, PI, in units of TJ km^−1^, as:

2 $$PI\,=\,{\sum }_{i}\left(\frac{{IKE}^{i}}{{Distance}^{i}}\times {t}_{S}^{i}\right)$$

The sum of ${t}_{s}^{i}$ is the total timespan in hours (${t}_{S}$). PI thus represents the cumulative energy of a cyclone during the timespan. We calculated PI as total PI (PI_T_) using IKE_T_ and sub-quadrant PI (PI_Q_) using IKE_Q_, respectively.

### Tropical cyclones-induced excess litter (EXS)

We assessed the annual NPP_L_ anomaly over the study period (2001–2018) to identify years with frequent cyclone impacts. Given the minimum cyclone impacts on SRE mangrove sites in the period 2001–2003, the average NPP_L_ over these three years was considered as baseline value to estimate the anomaly (Table. S1). The annual NPP_L_ anomaly was calculated as the difference between the total litterfall material collected in one year in the period 2004–2018 and the site-based annual NPP_L_ baseline (i.e., three-year averaged annual NPP_L_ for each site). The magnitude of this anomaly can be above (positive, surplus) or below (negative, deficit) the baseline; the concept is equivalent to the air temperature anomaly used in climate change studies^142^.

The EXS term represents the total amount of excess litterfall material that was deposited in a litter basket after cyclone impact in comparison to non-cyclone impact (i.e., 2001–2003 baseline). The value was calculated using the difference between the amount of the first samples collected after cyclone impact and its corresponding monthly baseline per basket. We defined the term R_EXS_ as the ratio of the EXS value to the basket-based annual baseline (i.e., three-year averaged annual NPP_L_ for each basket). The R_EXS_ value normalizes the differences for each cyclone and facilitates its comparison across all sites (Table 1). Because there was no sample collected between Hurricane Frances (September 5th) and Jeanne (September 26th) in 2004, we assumed that the EXS value was the result of the sum of PIs (see section above) for these cases and based on the litter samples collected on October 13th, 2004.

Using annual NPP_L_ estimates and the degree of canopy defoliation at each study site after Wilma’s impact in 2005, Danielson et al.^62^ used the SRS-4 excess litterfall value as a baseline to estimate how much litterfall in the baskets was removed due to the storm surge and high-speed wind impact on the other two sites (SRS-5 and SRS-6) where defoliation was > 50–90%. We followed Danielson et al.’s approach to calculate the EXS and R_EXS_ values for cyclone Wilma (2005) in all study sites (Table 1). This extrapolation was performed assuming the same cyclone strength across all sites based on Wilma’s wind field velocity and trajectory along the SRE^62^ (Fig. 2). In the case of cyclone Irma (Fig. 2), we assumed that the cyclone energy dissipated linearly from downstream (SRS-6) to the mid- (SRS-5) and upstream (SRS-4) regions since this storm followed a northbound trajectory, perpendicular to the SRE (Fig. 2)^88^. Since Irma passed closest to the SRS-6 site, the forest canopy was heavily defoliated (> 90%) indicating that most of the canopy material was exported. Although canopy defoliation at SRS-5 and SRS-4 was less evident compared to SRS-6, still excess litter produced by high winds was deposited in the litter baskets. Because only at SRS-6 partial litterfall in baskets were removed, we used a linear regression between observed R_EXS_ values at SRS-5 and SRS-4 and the distances from each study site to the SRE mouth to determine the potential R_EXS_ value at SRS-6 (Table 1). We then used this linearly estimated R_EXS_ to determine the total litterfall deposited at SRS-6 (i.e., “reconstructed” litterfall, see below) before export and removal by Irma’s storm surge.

### Litterfall exported by tidal exchange and storm surge

Litterfall can be exported through daily tidal cycles and storm surges caused by cyclones^13,74,110,143^. Due to the lack of direct measurements assessing the lateral flux at the boundary between the forest and adjacent coastal waters along the SRE during a tidal cycle, we used previously estimated export rates based on a soil organic matter and nutrient accumulation modeling study performed for the same study sites. This study considered the site-specific influence of hydroperiod, topography, and consumers activity^144^. These export rates assumed a site-specific percentage removal of soil litter accumulated on the soil surface by tidal exchange (SRS-4, SRS-5: 20%; SRS-6: 40%). We also used these rates to estimate the litter exported by the storm surge caused by weak and moderate cyclones. In the case of strong cyclones (Wilma and Irma), we observed litter export based on the material collected in the baskets at SRS-5 and SRS-6 during Wilma and only at SRS-6 in the case of Irma. Thus, litter export rates in those cases were estimated by the difference between the litter collected in the basket and the “reconstructed” total litter before being exported or removed by the storm surge (see above). Given the complex dynamics in assessing these export rates, these values are considered first-order estimations where 20% of the litterfall is assumed to be exported from the forest ground at SRS-4 during Wilma; this percentage was also used for the sites SRS-4 and SRS-5 in the case of Irma’s impact. Overall, we consider these export values as underestimates since the rates do not explicitly include the canopy materials “blown away” by high winds during the cyclone approach (Fig. 1).

### Statistical analysis

Since the EXS value represents excess litter caused by cyclones, we conducted one-sample upper tail Student’s t-test to determine which cyclone deposited excess litter (Prob > t; α = 0.05) that was significantly greater than zero (i.e., baseline litterfall 2001–2003). Regression analyses (α = 0.05) were performed to establish the relationship between R_EXS_ and the PI value (natural log transformed). Before the regression analyses, the normality of residuals was tested by the Shapiro–Wilk test (α = 0.05). We also performed linear regressions (α = 0.05) between R_EXS_ and other cyclone properties (SSHWS, wind speed, distance, and wind speed weighed by distance). All statistical analysis and graphs were performed using JMP Pro 15.2.1 (2019 SAS Institute Inc, Cary, NC, USA).

## Supplementary Information

Supplementary Figure S1.

Supplementary Figure S2.

Supplementary Figure S3.

Supplementary Tables.

## Data Availability

The datasets generated during and/or analyzed during the current study are available from the corresponding author on reasonable request.
