# Supplementary material for: Tropical cyclones cumulatively control regional carbon fluxes in Everglades mangrove wetlands (Florida, USA)
Source: Sci Rep. 2021 Jul 6;11:13927. doi: 10.1038/s41598-021-92899-1 (PMC8260777; doi:10.1038/s41598-021-92899-1)
Supplement: Supplementary file 1 — Supplementary Figure S1. [file 41598_2021_92899_MOESM1_ESM.docx]

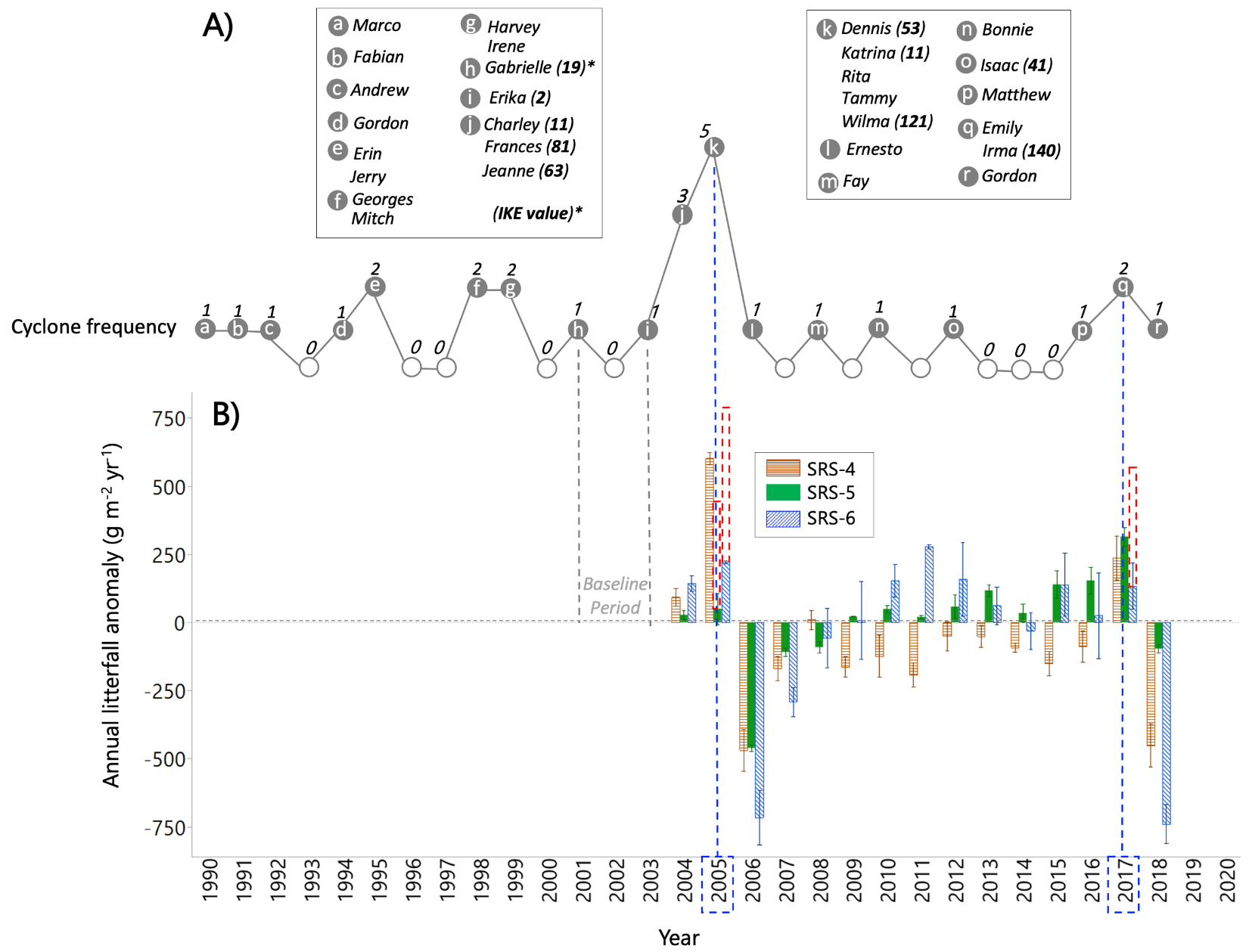


Figure S1. A) Annual frequency of tropical cyclones passing within 300-km (162-nautical miles) radii from the mangrove study sites along the Shark River Estuary (*center: SRS-5: 25°22'37.20"N, 81° 1'55.20"W*) in the period 1990-2018 (see Figure 2 for sites location). B) Annual litterfall dry mass anomaly (g m^-2^ yr^-1^) per site from 2004 - 2018. The anomaly values peaked in 2005 and 2017 (blue dash line) corresponding to large impacts by Wilma (k) and Irma (q). Red-dash boxes on bars at SRS-5 and SRS-6 in 2005 and at SRS-6 in 2017 indicate the litter mass collected in baskets that was potentially exported by storm surges (see Methods for flux estimates). The integrated kinetic energy (IKE_T_) is shown in parentheses in the tropical cyclone legend. Notice that the litterfall baseline period used to calculated the anomalies was determined by the litterfall collected monthly from 2001-2003; see Methods section.
