# Supplementary material for: Tropical cyclones cumulatively control regional carbon fluxes in Everglades mangrove wetlands (Florida, USA)
Source: Sci Rep. 2021 Jul 6;11:13927. doi: 10.1038/s41598-021-92899-1 (PMC8260777; doi:10.1038/s41598-021-92899-1)
Supplement: Supplementary file 2 — Supplementary Figure S2. [file 41598_2021_92899_MOESM2_ESM.docx]

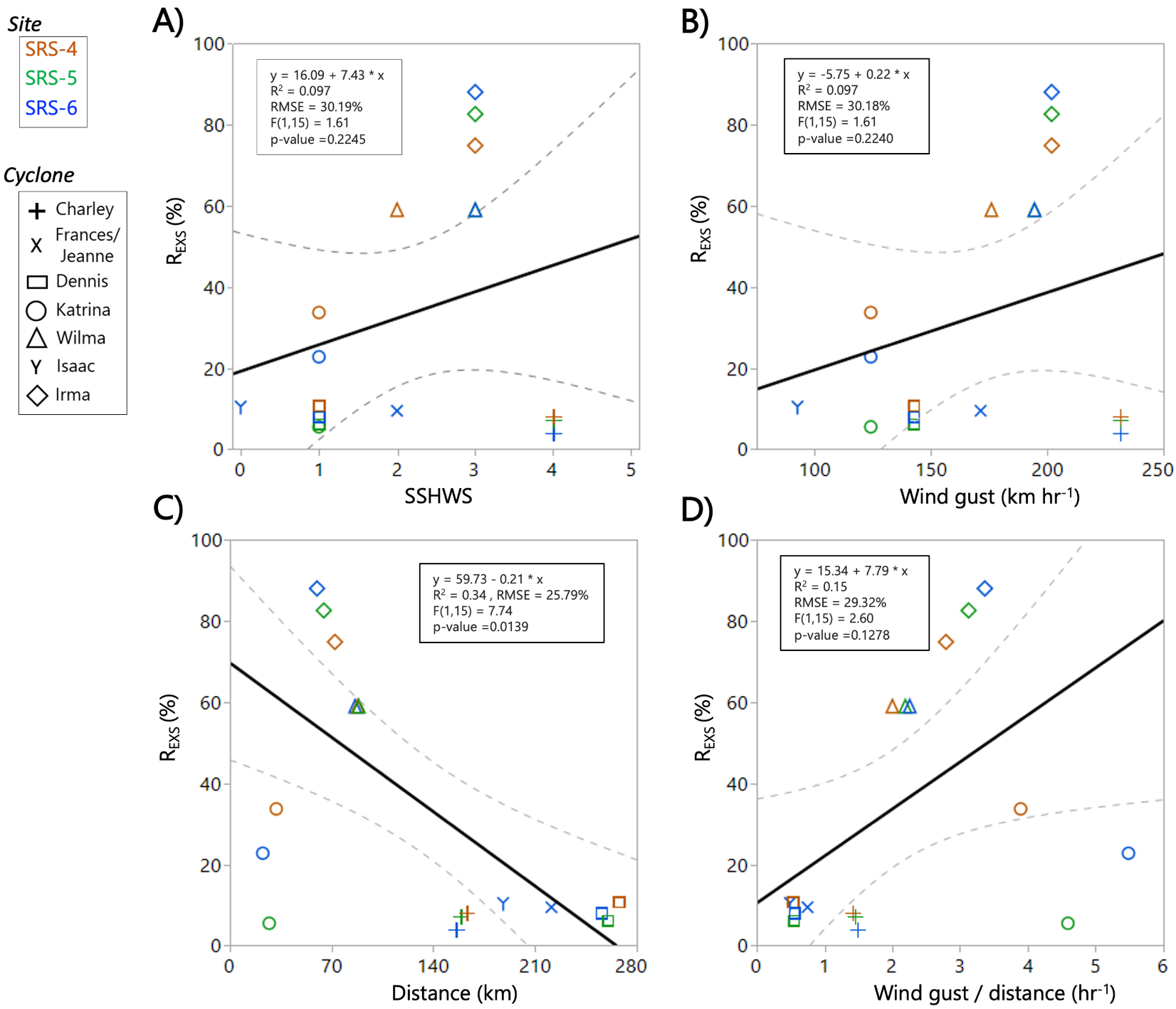


**Figure S2.** Regression models between different cyclone physical properties and R_EXS_ values; this variable is the ratio of the EXS value to the annual litterfall baseline. EXS is calculated using the difference between the amount of litterfall measured after cyclone impact and its corresponding monthly baseline (see Methods for details). **A**) Saffir-Simpson Hurricane Wind Scale (SSHWS, Category 1-5); **B)** wind gust when the cyclone was closest to the study sites; **C)** the shortest distance when the cyclone approached study site; **D**) the wind speed weighted by shortest distance to the study site. The dashed curve indicates the 95% confidence interval.
