# Supplementary material for: Tropical cyclones cumulatively control regional carbon fluxes in Everglades mangrove wetlands (Florida, USA)
Source: Sci Rep. 2021 Jul 6;11:13927. doi: 10.1038/s41598-021-92899-1 (PMC8260777; doi:10.1038/s41598-021-92899-1)
Supplement: Supplementary file 3 — Supplementary Figure S3. [file 41598_2021_92899_MOESM3_ESM.docx]

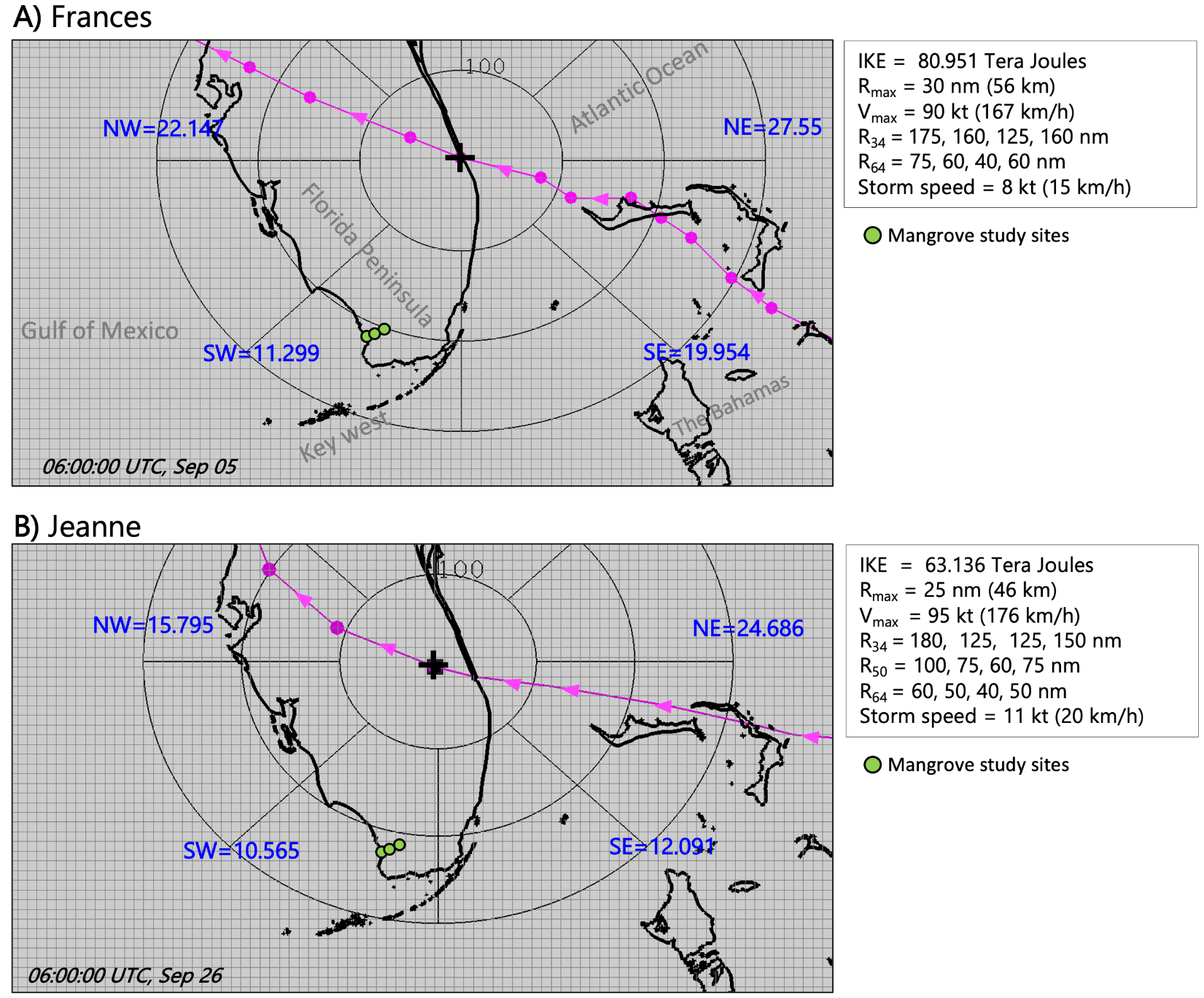


**Figure S3.** Integrated Kinetic Energy (IKE) values from tropical cyclone’s four quadrants: northeast (NE), southeast (SE), southwest (SW), and northwest (NW). **A)** Frances (Category 2) at 06:00 UTC on September 5, 2004 and **B**) Jeanne (Category 2) at 06:00 UTC on September 26, 2004. (**+**) = cyclone center; gray concentric circles delineate the 100-, 200-, and 300-km radii. Pink curve is the cyclone path and forward motion. Green dots mark the study site locations. See Methods for data and information sources.
