# Supplementary material for: Tropical cyclones cumulatively control regional carbon fluxes in Everglades mangrove wetlands (Florida, USA)
Source: Sci Rep. 2021 Jul 6;11:13927. doi: 10.1038/s41598-021-92899-1 (PMC8260777; doi:10.1038/s41598-021-92899-1)
Supplement: Supplementary file 4 — Supplementary Tables. [file 41598_2021_92899_MOESM4_ESM.docx]

SUPPLEMENTARY TABLES

Table S1. List of historical tropical cyclones (1990-2018) passing within 300-km radii (162 nautical miles) from the study site SRS-5 *(25°22'37.20"N, 81° 1'55.20"W*) in the Shark River Estuary (see Figure 2). The Saffir-Simpson Hurricane Wind Scale (SSHWS) ranges from Category 1 to 5. The values “0” and “-1” in the SSHWS column indicate that the cyclone was degraded to Tropical Storm (0) or Tropical Depression (-1) when passing within the 300-km radii. Wind gust, SSHWS, and translation speed values were recorded while the cyclone was the closest to study sites. See Methods for further information and calculations.

| **Cyclone Name** | **Year** | | **Month/Day** | | **Distance to SRS-5 (km)** | | **Wind Speed**  **(km h^-1^)** | | **SSHWS** | | **Translation Speed (km h^-1^)** | |
| --- | --- | --- | --- | --- | --- | --- | --- | --- | --- | --- | --- | --- |
| *1)* Marco | 1990 | | October 10 | | 149 | | 83.25 | | 0 | | 12.95 | |
| *2*) Fabian | 1991 | | October 16 | | 106 | | 74.00 | | 0 | | 31.45 | |
| *3)* Andrew | 1992 | | August 24 | | 30 | | 212.75 | | 4 | | 31.45 | |
| *4)* Gordon | 1994 | | November 15 | | 110 | | 83.25 | | 0 | | 12.95 | |
| *5)* Erin | 1995 | | August 02 | | 238 | | 138.75 | | 1 | | 27.75 | |
| *6)* Jerry |  | | August 23 | | 168 | | 59.20 | | -1 | | 24.05 | |
| *7)* Georges | 1998 | September 25 | | 125 | | 166.50 | | 2 | | 24.05 | |  |
| *8)* Mitch |  | | November 05 | | 126 | | 101.75 | | 0 | | 53.65 | |
| *9)* Harvey | 1999 | | September 21 | | 75 | | 92.50 | | 0 | | 35.15 | |
| *10)* Irene |  | | October 15 | | 11 | | 129.50 | | 1 | | 16.65 | |
| *11)* Gabrielle | 2001 | | September 14 | | 238 | | 101.75 | | 0 | | 29.60 | |
| *12)* Erika | 2003 | | August 14 | | 254 | | 64.75 | | 0 | | 38.85 | |
| *13)* Charley | 2004 | | August 13 | | 159 | | 231.25 | | 4 | | 33.30 | |
| *14)* Frances |  | | September 05 | | 219 | | 166.50 | | 2 | | 5.55 | |
| *15)* Jeanne |  | | September 26 | | 218 | | 175.75 | | 2 | | 20.35 | |
| *16)* Dennis | 2005 | | July 09 | | 260 | | 142.45 | | 1 | | 22.20 | |
| *17)* Katrina |  | | August 26 | | 27 | | 120.25 | | 1 | | 16.65 | |
| *18)* Rita |  | | September 20 | | 174 | | 157.25 | | 2 | | 20.35 | |
| *19)* Tammy |  | | October 05 | | 252 | | 64.75 | | 0 | | 18.50 | |
| *20)* Wilma |  | | October 24 | | 89 | | 194.25 | | 3 | | 48.10 | |
| *21)* Ernesto | 2006 | | August 30 | | 25 | | 74.00 | | 0 | | 14.80 | |
| *22)* Fay | 2008 | | August 19 | | 78 | | 101.75 | | 0 | | 12.95 | |
| *23)* Bonnie | 2010 | | July 23 | | 48 | | 55.50 | | -1 | | 27.75 | |
| *24)* Isaac | 2012 | | August 26 | | 190 | | 92.50 | | 0 | | 22.20 | |
| *25)* Matthew | 2016 | | October 07 | | 251 | | 212.75 | | 4 | | 22.20 | |
| *26)* Emily | 2017 | | August 01 | | 238 | | 55.50 | | -1 | | 14.80 | |
| *27)* Irma |  | | September 10 | | 65 | | 201.65 | | 3 | | 20.35 | |
| *28)* Gordon | 2018 | | September 03 | | 20 | | 85.10 | | 0 | | 25.90 | |

Table S2. Variation in tropical cyclones induced excess litterfall values (EXS, g m^-2^) in mangrove study sites (SRS-4, SRS-5 and SRS-6) along the Shark River Estuary, Everglades, South Florida, USA in the period 2004-2018. Student’s t-test p-values followed by “***” indicating mean EXS (± SE) are signiﬁcantly greater than zero (p < 0.05). Shapiro-Wilk test p-values followed by “***” indicating a non-normal distribution of EXS (p < 0.05). Notice there was no litterfall sample collected between cyclones Frances and Jeanne in 2004 or between Rita and Tammy in 2005. See Methods.

| **Year** | **Cyclone** | **Passage Date** | **Study Site** | **Sampling date** | **EXS (g m^-2^)** | **Student’s t-test (Prob > t)** | **Shapiro-Wilk test** |
| --- | --- | --- | --- | --- | --- | --- | --- |
| 2004 | Charley | August 13 | SRS-4 | August 16 | 69.6 ± 12.6 | 0.0002*** | 0.1373 |
|  |  |  | SRS-5 |  | 61.2 ± 25.8 | 0.0032*** | 0.0895 |
|  |  |  | SRS-6 |  | 40.7 ± 5.8 | 0.0003*** | 0.5848 |
|  | Frances/  Jeanne | September 5/  September 26 | SRS-4 | October 13 | 22.5 ± 22.3 | 0.1704 | 0.2200 |
|  |  |  | SRS-5 |  | 25.6 ± 25.1 | 0.1666 | 0.0252*** |
|  |  |  | SRS-6 |  | 100.6 ± 21.3 | 0.0065*** | 0.0242*** |
| 2005 | Dennis | July 9 | SRS-4 | July 21 | 92.6 ± 33.4 | 0.0003*** | 0.6338 |
|  |  |  | SRS-5 |  | 53.8 ± 36.6 | 0.0149*** | 0.2691 |
|  |  |  | SRS-6 |  | 88.5 ± 25.9 | <0.0001*** | 0.9915 |
|  | Katrina | August 26 | SRS-4 | September 13 | 293.7 ± 67.6 | 0.0002*** | 0.5587 |
|  |  |  | SRS-5 |  | 50.3 ± 5.9 | 0.0509 | 0.2084 |
|  |  |  | SRS-6 |  | 240.6 ± 15.5 | 0.0001*** | 0.2163 |
|  | Rita/  Tammy | September 20/  October 5 | SRS-4 | October 11 | -27.9 ± 11.4 | 0.9818 | 0.8804 |
|  |  |  | SRS-5 |  | -25.9 ± 9.6 | 0.9880 | 0.3547 |
|  |  |  | SRS-6 |  | -57.3 ± 9.7 | 0.9999 | 0.9672 |
|  | Wilma | October 24 | SRS-4 | November 1 | 511.6 ± 36.4 | <0.0001*** | 0.8332 |
|  |  |  | SRS-5 |  | 487.7 ± 16.1 | <0.0001*** | 0.6083 |
|  |  |  | SRS-6 |  | 629.5 ± 38.1 | <0.0001*** | 0.8303 |
| 2012 | Isaac | August 27 | SRS-4 | September 10 | 8.7 ± 21.0 | 0.3437 | 0.0816 |
|  |  |  | SRS-5 |  | 6.3 ± 10.8 | 0.2865 | 0.6083 |
|  |  |  | SRS-6 |  | 104.7 ± 37.2 | 0.0102*** | 0.3893 |
| 2017 | Irma | September 10 | SRS-4 | October 10 | 644.8 ± 74.9 | <0.0001*** | 0.3084 |
|  |  |  | SRS-5 |  | 655.4 ± 64.2 | <0.0001*** | 0.4566 |
|  |  |  | SRS-6 |  | 940.9 ± 56.9 | <0.0001*** | 0.8303 |

**Table S3**. Particulate organic carbon (POC) export as litter flux (Litter-POC; g C m^-2^ yr^-1^) and litterfall net primary production (NPP_L_;

g C m^-2^ yr^-1^) variation in a range of mangrove ecotypes (modified from *Adame and Lovelock 2011*). A factor of 0.44 was used to convert from g dry mass m^-2^ yr^-1^ to g C m^-2^ yr^-1^ (see Methods).

| **Location** | **Site** | **Mangrove ecotype** | **Average tidal amplitude (m)** | | **Litter-POC flux (g C m^-2^ yr^-1^)** | | **NPP_L_**  **(g C m^-2^ yr^-1^)** | | **% of Litter-POC contribution to NPP_L_** | | **Source** | | |
| --- | --- | --- | --- | --- | --- | --- | --- | --- | --- | --- | --- | --- | --- |
| Shark River Estuary, Florida, USA | SRS-4 | Riverine | |  | | -71 | | 381 | | 19 | | This study |  |
|  | SRS-5 | Riverine | |  | | -84 | | 365 | | 23 | | This study |  |
|  | SRS-6 | Riverine | | 0.75 | | -205 | | 464 | | 44 | | This study |  |
| Florida, USA | North River |  | | 0.8 | | -292 | | 387 | | 75 | | Odum & Heald (1972, 1974) |  |
|  | Florida |  | | 0.8 | | -186 | |  | |  | | Healld (1969), Odum & Heald (1974) |  |
|  | South Florida |  | | 0.8 | | -438 | |  | |  | | Healld (1971), Odum & Heald (1974) in Miller (1979) |  |
|  | Rookery Bay | Basin | | 0.1 | | -198 | | 213 | | 93 | | Lugo & Snedaker (1974), Twilley et al. (1986) |  |
| Mexico | El verde largoon |  | | 1.5 | | -492.8 | | 484 | | 102 | | Flores-Verdugoet et al. (1987) |  |
| Brazil | Itacuruca | Fringe | | 0.8 | | ~-3.9 | | 297 | | 1 | | Adaime (1985), Rezende (1988), Lacerda et al. (1995), Silva et al. (1998), Rezende et al. (2007) |  |
| Puerto Rico | Magueyes Is. |  | | 0.3 | | ~-401.5 | | 319 | | 125 | | Golley et al. (1962) |  |
| Ecuador | Guayas River | Riverine | | 4.0 | | -340.1 | | 367 | | 93 | | Twilley et al. (1997) |  |
| Australia | Darwin |  | | 3.7 | | -332 | | 302 | | 110 | | Woodroffe et al. (1988) |  |
|  | Townsville |  | | 2.2 | | -194 | | 422 | | 46 | | Robertson & Daniel (1989) |  |
|  | Townsville |  | | 2.2 | | -252 | | 422 | | 60 | | Robertson & Daniel (1989) |  |
|  | Ingham |  | | 2.2 | | -107 | | 422 | | 25 | | Robertson & Daniel (1989) |  |
|  | Coral Creek |  | | 1.8 | | -365 | | 392 | | 93 | | Boto & Bunt (1981), Ayukai et al. (1998), Boto & Wellington (1988) |  |
|  | Kuala Selangor | Fringing/Riverine | | 2.8 | | -122.6 | | 311 | | 39 | | Mahmood et al. (2005) |  |
|  | Mattang | Riverine | | 2.7 | | -176 | | 336 | | 52 | | Gong et al. (1984), Gong & Ong (1990) |  |
| New Zealand | Tuff Crater | Fringe/Scrub | | 1.3 | | ~-2.2 | | 259 | | <1 | | Woodroffe (1982), Woodroffe (1985a) |  |
| Hong Kong | Mai Po |  | | 1.4 | | -3.7 | | 487 | | <1 | | Lee (1989) |  |
| India | Mandovi-Zuari |  | | 1.3 | | -0.1 | | 558 | | <1 | | Quasim & Sen Gupta (1981), Wafar et al. (1997) |  |
| Thailand | Klon Ngao |  | | 2.4 | | ~-1.0 | | 295 | | <1 | | Wattayakorn et al. (1990) |  |
| South Africa | Mngazana | Fringe | | 0.48 | | -131.6 | | 246 | | 53 | | Rajkaran & Adams (2007) |  |
